# Accuracy of Artificial Intelligence Versus Clinicians in Real-Life Case Scenarios of Retinopathy of Prematurity

**DOI:** 10.7759/cureus.78597

**Published:** 2025-02-05

**Authors:** Akash Belenje, Dhanush Pandya, Subhadra Jalali, Padmaja K Rani

**Affiliations:** 1 Srimati Kanuri Santhamma Center for Vitreo-Retinal Diseases, Anant Bajaj Retina Institute, Kallam Anji Reddy Campus, L V Prasad Eye Institute, Hyderabad, IND

**Keywords:** artificial intelligence, chatgpt, real-life case scenarios, retinopathy of prematurity, vitroretinal fellowship trainees

## Abstract

Objective

The aim of this study was to compare the accuracy of ChatGPT artificial intelligence (AI) with clinicians in real-life case scenarios related to retinopathy of prematurity (ROP).

Methods

This was a prospectively conducted study with a real-life case scenario-based questionnaire with multiple-response answers. Thirteen clinicians, including eight vitreoretinal fellowship trainees (with less than two years of experience in the management of ROP) and five ROP experts (with more than three years of experience in the management of ROP), were given 10 real-life case scenarios in ROP. The majority of responses from trainees and ROP experts were compared with the ChatGPT AI-generated responses. The ChatGPT exercise was repeated for both versions 3.5 and 4.0 more than a month apart on May 29, 2024, and July 18, 2024, to check for the majority of AI response consistency. For each real-life case scenario, the majority of clinician responses were compared with the majority of AI responses for agreement.

Results

ChatGPT answered nine cases correctly (90%), outperforming the fellowship trainees (77.5%, i.e., 62 correct responses out of 80). The accuracy of ROP experts was highest at 96% (i.e., 48 correct responses out of 50). There was substantial agreement between the majority of responses of clinicians and the ChatGPT responses, with a Cohen’s kappa of 0.80.

Conclusion

The ChatGPT AI model showed substantial agreement with the majority of clinician responses and performed better than vitreoretinal fellowship trainees. ChatGPT AI presents promising new software tools that can be explored further for use in real-life case scenarios in ROP. A more accurate prompt mentioning the type of screening guidelines can promote more accurate answers by ChatGPT as per the requested guidelines.

## Introduction

Retinopathy of prematurity (ROP) is the leading cause of preventable blindness in infants worldwide [1]. It is a disorder of immature retinal vasculature in preterm delivered babies [2]. It is a time-bound vasoproliferative disease, and important neonatal risk factors include early gestational age, low birth weight, suboptimal antenatal care, suboptimal neonatal care with supplementation of unblended unmonitored 100% oxygen, respiratory distress, apnea, poor weight gain, neonatal sepsis, and anemia [3-4]. The newer advances in pediatric retinal imaging have helped the clinician in better understanding and diagnosis of ROP [5-6]. However, the wide spectrum of clinical presentation and severity of the disease still pose a significant challenge to clinicians in the appropriate management of ROP.

Widefield imaging, tele ophthalmology, and artificial intelligence (AI) have reduced the screening and diagnostic workload of ophthalmologists [5-8]. Use of AI in the real world is predominantly focused on large volumes of data acquisition in building algorithms for accurate validation of clinical diagnosis [9,10]. AI models have been used in the past for the detection and grading of high-incidence ophthalmological diseases including cataract, glaucoma, diabetic retinopathy, age-related macular degeneration, retinal vein occlusion, and ROP [11]. ChatGPT (OpenAI Inc., San Francisco, CA, USA) is an AI-generated language model, with the ability to process and reply to text, images, and graphical inputs into human-like conversation outputs [12]. Recent studies have explored the reliability and accuracy of ChatGPT AI in providing information, screening, and interpretation of ophthalmic diseases in various case scenarios [13-15]. ROP is one of the most unpredictable vascular diseases posing screening, diagnosis, and management dilemma to treating ophthalmologists. Focused use of ChatGPT AI model in real-life ROP case scenarios in comparison to clinician response has never been explored before. Hence, the objective of our prospective study is to compare the accuracy of ChatGPT AI with clinicians in real-life case scenarios related to ROP.

## Materials and methods

This was a prospective cross-sectional study with real-life case scenario-based questionnaire with multiple-response answers. The study was approved by the Institute Review Board (LEC-BHR-P-09-22-926) and adhered to the tenets of the Declaration of Helsinki. Ten real-life case scenarios pertaining to screening, diagnosis, and treatment of ROP were included. For each case scenario, three answer responses were given, and only one best response that best addressed the case scenario was to be selected. Exclusion criteria include an image-based questionnaire in ROP; multiple-response answers to a single question is not considered, and only one best response to a single question is considered. It was a multicenter study across three states of southern India. All the 10 real-life case scenario questionnaires were given to eight vitreoretinal fellowship trainees with less than two years of experience in the screening and management of ROP. The questionnaires were then given to five vitreoretinal consultant ROP experts with more than three years of experience in the screening and management of ROP. Informed consent was taken from all thirteen clinicians participating in the study. The responses from all 13 clinicians were noted for each question on May 24, 2024. The exact set of questionnaires was later fed to ChatGPT versions 3.5 and 4.0 on May 29, 2024. The ChatGPT exercise was repeated for both versionss 3.5 and 4.0 more than a month apart on July 18, 2024, to check for the majority of AI response consistency. The accuracy of ChatGPT in comparison to clinician response was analyzed. For each real-life case scenario, the majority of clinician responses were compared with the majority of AI responses for agreement.

Below are a few examples of case scenarios with ChatGPT responses:

Example 1 (prompt)

Case Scenario

A preterm baby was delivered at 26 weeks of gestational age with a birth weight of 1,200 grams and postmenstrual age of 30 weeks. Both eyes showed aggressive ROP with dilated and tortuous vessels in half zone with incomplete foveal vascularization.

The answer must be only one of the following:

1. Both eyes are treated with an intravitreal anti-vascular endothelial growth factor (VEGF) agent

 2. Both eyes are treated with a laser indirect ophthalmoscope

3. No treatment is required.

ChatGPT Response

Aggressive ROP, particularly with the described vascular abnormalities, is at high risk for leading to severe outcomes, including retinal detachment. The treatment options typically include the following:

Intravitreal anti-VEGF agent: This treatment can be particularly effective in aggressive cases of ROP where rapid action may be needed to prevent progression. Anti-VEGF agents can cause regression of neovascularization and decrease vascular activity.

Laser indirect ophthalmoscope: This is a more traditional treatment that involves using a laser to ablate peripheral areas of the retina that are not fully vascularized. This treatment prevents further progression of the disease by reducing the drive for abnormal vessel growth.

Given the specifics of this scenario with aggressive ROP showing dilated and tortuous vessels and incomplete foveal vascularization, and considering the need for rapid and effective intervention:

1. Both eyes are treated with an intravitreal anti-VEGF agent.

This option is chosen because anti-VEGF treatments are particularly effective for aggressive forms of ROP, as they can rapidly reduce the activity of the disease, helping in stabilizing the condition quicker than laser treatment, which is crucial given the severity described.

Example 2 (prompt)

Case Scenario

A preterm baby was delivered at 28 weeks of gestational age with a birth weight of 1,400 grams and a postmenstrual age of 40 weeks. Both eyes showed a well-dilated pupil with zone 2 stage 4B tractional retinal detachment.

1. Both eyes are treated with an intravitreal anti-VEGF agent alone.

2. Both eyes are treated with a laser indirect ophthalmoscope alone.

3. Both eyes immediate lens sparing vitrectomy with endo laser.

ChatGPT Response

Zone 2, stage 4B tractional retinal detachment indicates that the retina has already begun detaching due to the traction from fibrovascular membranes. This is a severe and advanced stage of ROP, which requires prompt and effective surgical treatment to attempt reattachment of the retina and prevent permanent vision loss. Immediate lens-sparing vitrectomy with endo laser involves the surgical removal of the vitreous that is tugging on the retina and the application of laser to the retina to secure it in place. This approach is suitable for managing severe tractional detachments such as stage 4B, as it directly addresses the mechanical aspect of the detachment.

Given these considerations, the correct treatment approach for this scenario would be:

3. Both eyes immediate lens sparing vitrectomy with endo laser.

Example 3 (prompt)

Case Scenario

A preterm baby was delivered at 26 weeks of gestational age with a birth weight of 1,400 grams and a postmenstrual age of 30 weeks. Both eyes fundus showed aggressive ROP in zone 1. What is the dose of intravitreal bevacizumab injected?

The answer must be only one of the following:

 1. 1.25 mg in 0.05 mL

 2. 0.625 mg in 0.025 mL

 3. 1.25 mg in 0.025 mL

ChatGPT Response

For the treatment of aggressive ROP in zone 1 using intravitreal bevacizumab, the commonly administered dose according to established guidelines and clinical studies (such as the BEAT-ROP study) is:

2. 0.625 mg in 0.025 mL per eye.

## Results

Statistical analysis

SAS (Statistical Analysis System, Version 3.81, Enterprise Edition, SAS Institute Inc., Cary, NC, USA) software was used for the analysis. Intra-observer and inter-observer analysis was used for Cohen’s kappa analysis. The consistency of ChatGPT responses for both versions 3.5 and 4.0 is shown in Table 1. Cohen's kappa was 1.0, which indicates perfect agreement between the responses for both the versions of ChatGPT (3.5 and 4.0) from May 29 and July 18. The chi-square test p-value was 0. 0855, which indicates that there is no significant difference in the responses between the two dates. This p-value is greater than the common significance level of 0.05, indicating that there is no significant difference in the distribution of responses between the two dates.

**Table 1:** ChatGPT responses consistency for both versions 3.5 and 4.0 VEGF, vascular endothelial growth factor

Case scenario	Response (May 29)	Response (July 18)	Cohen’s kappa
1	Screening must be done immediately	Screening must be done immediately	1
2	Screening must be done within 1 month	Screening must be done within 1 month	1
3	Treatment must be done immediately	Treatment must be done immediately	1
4	Observe and review in 2 weeks	Observe and review in 2 weeks	1
5	Observe and review in 1 week	Observe and review in 1 week	1
6	Intravitreal anti-VEGF	Intravitreal anti-VEGF	1
7	Immediate lens-sparing vitrectomy	Immediate lens-sparing vitrectomy	1
8	Posterior zone 2 definition incorrect	Posterior zone 2 definition incorrect	1
9	1.25 mg in 0.05 mL	1.25 mg in 0.05 mL	1
10	Treatment must be done immediately	Treatment must be done immediately	1

Cohen’s kappa agreement between majority of clinician responses and ChatGPT majority of AI-generated responses for each question is shown in Table 2. ChatGPT answered nine questions correctly (90%), outperforming the fellowship trainees (77.5%, i.e., 62 correct responses out of 80). The accuracy of ROP experts was highest at 96% (i.e., 48 correct responses out of 50). There was substantial agreement between the majority of responses of clinicians and the ChatGPT response, with an average Cohen’s kappa of 0.80.

**Table 2:** Agreement between majority of clinician responses and majority of ChatGPT AI-generated responses ROP, retinopathy of prematurity; VR, vitreoretinal

Question number	VR fellow response (8 VR fellows)	ROP expert response (5 ROP experts)	Majority of clinician responses (13 clinicians)	Majority of ChatGPT 3.5/4.0 responses (May 29, 2024, and July 18, 2024)	Cohen’s kappa
1	6 correct, 2 wrong	5 correct	11 correct, 2 wrong; 84.6% agreement	4 correct; 100% agreement	0.82
2	5 correct, 3 wrong	4 correct, 1 wrong	9 correct, 4 wrong; 69.2% agreement	4 correct; 100% agreement	0.54
3	7 correct, 1 wrong	5 correct	12 correct, 1 wrong; 92.3% agreement	4 correct; 100% agreement	0.88
4	7 correct, 1 wrong	5 correct	12 correct, 1 wrong 92.3% agreement	4 correct; 100% agreement	0.88
5	6 correct, 2 wrong	5 correct	11 correct, 2 wrong; 84.6 % agreement	4 correct; 100% agreement	0.82
6	8 correct	5 correct	13 correct 100% agreement	4 correct; 100% agreement	1.0
7	8 correct	5 correct	13 correct 100% agreement	4 correct; 100% agreement	1.0
8	3 correct, 5 wrong	4 correct, 1 wrong	7 correct, 6 wrong 53.8% agreement	4 wrong; 0% agreement	0.08
9	5 correct, 3 wrong	5 correct	10 correct, 3 wrong 76.92% agreement	4 correct; 100% agreement	0.74
10	7 correct,1 wrong	5 correct	12 correct, 1 wrong 92.3% agreement	4 correct; 100% agreement	0.88
Overall	62 correct responses out of 80; 77.5% agreement	48 correct responses out of 50; 96% agreement	110 correct responses out of 130; 76.2% agreement	36 correct responses out of 40; 90% agreement	0.80

ChatGPT could not give an accurate answer to only one question, and the case scenario is described below.

Prompt

Key components of International Classification of Retinopathy of Prematurity (ICROP), 3rd edition, include all the below except:

The answer must be only one of the following:

1. Posterior zone 2 begins at the margin between zone 1 and extends into zone 2 for 2-disc diameters.

2. The term notch is used to describe an incursion by ROP lesion of 1-2 clock hours into a more posterior zone.

3. The term aggressive posterior retinopathy of prematurity (APROP) is used to describe severe ROP.

ChatGPT response

In the ICROP, 3rd edition, various terms and zones are specifically defined to guide the diagnosis and treatment of ROP. Understanding these definitions is crucial for the correct management of the disease. Let's look at the statements provided:

"Posterior zone 2 begins at the margin between zone 1 and extends into zone 2 for 2-disc diameters." This statement is incorrect as per the ICROP definitions. In the classification, zone 2 starts at the edge of zone 1 (defined as twice the distance from the optic disc to the macula, circular in shape) and extends outwards to the nasal ora serrata. It does not describe an extension "into zone 2 for 2-disc diameters."

"The term notch is used to describe an incursion by ROP lesion of 1-2 clock hours into a more posterior zone." This statement is accurate. The term "notch" in the context of ROP is used to describe the localized advancement of the disease into a more posterior zone, typically involving 1-2 clock hours.

"The term aggressive posterior retinopathy of prematurity (APROP) is used to describe severe ROP." This statement is also accurate. APROP is a term used in ROP classification to describe a particularly severe, rapidly progressing form of the disease that is characterized by its posterior location and poor differentiation between vascularized and avascular retina.

Given this analysis, the answer to the query about which statement is not included in the third edition of the ICROP is:

1. Posterior zone 2 begins at the margin between zone 1 and extends into zone 2 for 2-disc diameters.

The ICROP, 3rd edition, has replaced the terminology APROP with A-ROP (aggressive retinopathy of prematurity), and the correct response is three. This was not correctly identified by ChatGPT.

## Discussion

Previous studies have used ChatGPT responses in real-life case scenarios in diabetic retinopathy screening, interpretation of clinical ophthalmological images, and diagnosis of neuro-ophthalmology cases [14-16]. In our study, we have compared the accuracy of responses between ChatGPT versus vitreoretinal fellowship trainees versus ROP experts in various real-life case scenarios pertaining to ROP. We noticed that the ChatGPT response was more accurate when the type of screening and treatment guidelines were mentioned in the case scenario. An example is given below.

Prompt

A preterm baby was delivered at 30 weeks of gestational age with a birth weight of 1,500 grams and a postmenstrual age of 34 weeks. Both eyes showed ROP with a severity of zone 2, stage 3, plus disease.

According to the Early Treatment for Retinopathy of Prematurity (ETROP) study, the answer must be only one of the following:

1. Treatment must be done immediately.

2. Observe and review in two weeks.

3. No treatment is required.

ChatGPT response

For a preterm baby born at 30 weeks of gestational age, weighing 1,500 grams, and now at a postmenstrual age of 34 weeks, with both eyes showing retinopathy of prematurity (ROP) at zone 2, stage 3, plus disease, the treatment guidelines are specific. According to the Early Treatment for Retinopathy of Prematurity (ETROP) study, this condition requires immediate intervention.

Zone 2, stage 3, plus disease is considered severe enough to warrant urgent treatment because "plus disease" indicates progressive vascular dilation and tortuosity associated with worsening disease, which significantly increases the risk of poor outcomes, including retinal detachment.

Therefore, the correct response based on the scenario provided would be:

1. Treatment must be done immediately.

This immediate treatment typically involves laser therapy or anti-VEGF injections to prevent the progression of the disease and potentially serious complications.

Similarly, ChatGPT response was more accurate when the Indian ROP guidelines for screening or ETROP guidelines for treatment is mentioned in the questionnaire.

The description of the answers was much more in detail in the ChatGPT 4.0 version when compared to ChatGPT 3.5 version. However, both the versions were consistent with perfect agreement when checked on two different dates six weeks apart (May 29 and July 18). ChatGPT outperformed the vitreoretinal trainees and had substantial agreement with majority of clinician responses. ChatGPT AI can assist general ophthalmologists and ROP trainees in screening babies for ROP and referring them to higher-level treatment centers when necessary.

The strengths of the study are that the study questionnaire and case scenarios were constructed and validated by two ROP experts with more than five years of experience. The study was conducted among ROP specialists, including trainees, to understand the need for and utility of the ChatGPT tool in real-world scenarios. The study validated both the free and paid versions of the ChatGPT tool. The limitations of the study were the small sample size and that there were no ROP image-based questionnaires and hence we could not compare ChatGPT performance with clinicians in image-based case scenarios. ChatGPT AI is a language model and not yet robust in giving analysis for complex images of ROP. Hence, we have used ChatGPT responses only for real-life case scenario-based questionnaires in ROP. However, future versions of ChatGPT AI models with robust image analysis will be much more helpful in assisting general ophthalmologists and ROP trainees in screening babies for ROP.

Summary

What Was Known Before

AI models have been used in the past for the detection and grading of high-incidence ophthalmological diseases including cataract, glaucoma, diabetic retinopathy, age-related macular degeneration, retinal vein occlusion, and ROP. ChatGPT is an AI-generated language model, with the ability to process and reply to text, images, and graphical inputs into human-like conversation outputs.

What This Study Adds

The study was conducted among ROP specialists, including trainees, to understand the need for and utility of the ChatGPT tool in real-world scenarios. The ChatGPT AI model showed substantial agreement with the majority of clinician responses. ChatGPT AI presents promising new software tools that can be explored further for use in real-life case scenarios in ROP. A more accurate prompt mentioning the type of screening guidelines can promote more accurate answers by ChatGPT as per the requested guidelines.

## Conclusions

The ChatGPT AI model showed substantial agreement with the majority of clinician responses and performed better than vitreoretinal fellowship trainees. The ChatGPT responses are consistent with perfect agreement between the two versions (3.5 and 4.0) on different dates. A more accurate prompt mentioning the type of screening guidelines can promote more accurate answers by ChatGPT as per the requested guidelines. ChatGPT AI presents a promising new software tool that can be explored further for use in real-life case scenarios in ROP.

## References

[1] Solebo AL, Teoh L, Rahi J (2017). Epidemiology of blindness in children. Arch Dis Child.

[2] Ashton N, Ward B, Serpell G (1954). Effect of oxygen on developing retinal vessels with particular reference to the problem of retrolental fibroplasia. Br J Ophthalmol.

[3] Alajbegovic-Halimic J, Zvizdic D, Alimanovic-Halilovic E, Dodik I, Duvnjak S (2015). Risk factors for retinopathy of prematurity in premature born children. Med Arch.

[4] Kim SJ, Port AD, Swan R, Campbell JP, Chan RV, Chiang MF (2018). Retinopathy of prematurity: a review of risk factors and their clinical significance. Surv Ophthalmol.

[5] Belenje A, Reddy RU, Optom B, Agarwal K, Parmeswarappa DC, Jalali S (2023). Non-contact widefield neonatal retinal imaging for retinopathy of prematurity using the Clarus 700 high resolution true colour reflectance imaging. Eye (Lond).

[6] Belenje A, Reddy RU, Parmeswarappa DC, Padhi TR, Subbarao B, Jalali S (2024). Evaluation of optical coherence tomography biomarkers to differentiate favourable and unfavourable responders to intravitreal anti-vascular endothelial growth factor treatment in retinopathy of prematurity. Eye (Lond).

[7] Ramanathan A, Athikarisamy SE, Lam GC (2023). Artificial intelligence for the diagnosis of retinopathy of prematurity: a systematic review of current algorithms. Eye (Lond).

[8] Padhi TR, Bhunia S, Das T (2024). Outcome of real-time telescreening for retinopathy of prematurity using videoconferencing in a community setting in Eastern India. Indian J Ophthalmol.

[9] Gensure RH, Chiang MF, Campbell JP (2020). Artificial intelligence for retinopathy of prematurity. Curr Opin Ophthalmol.

[10] Honavar SG (2022). Artificial intelligence in ophthalmology - machines think!. Indian J Ophthalmol.

[11] Du XL, Li WB, Hu BJ (2018). Application of artificial intelligence in ophthalmology. Int J Ophthalmol.

[12] Ting DS, Tan TF, Ting DS (2024). ChatGPT in ophthalmology: the dawn of a new era?. Eye (Lond).

[13] Cappellani F, Card KR, Shields CL, Pulido JS, Haller JA (2024). Reliability and accuracy of artificial intelligence ChatGPT in providing information on ophthalmic diseases and management to patients. Eye (Lond).

[14] Mihalache A, Huang RS, Popovic MM (2024). Accuracy of an artificial intelligence chatbot's interpretation of clinical ophthalmic images. JAMA Ophthalmol.

[15] Gopalakrishnan N, Joshi A, Chhablani J (2024). Recommendations for initial diabetic retinopathy screening of diabetic patients using large language model-based artificial intelligence in real-life case scenarios. Int J Retina Vitreous.

[16] Madadi Y, Delsoz M, Lao PA, Fong JW, Hollingsworth TJ, Kahook MY, Yousefi S (2024). ChatGPT assisting diagnosis of neuro-ophthalmology diseases based on case reports [Preprint]. J Neuroophthalmol.

